# Electronic polarization stabilizes tertiary structure prediction of HP-36

**DOI:** 10.1007/s00894-014-2195-7

**Published:** 2014-04-09

**Authors:** Li L. Duan, Tong Zhu, Qing G. Zhang, Bo Tang, John Z. H. Zhang

**Affiliations:** 1 College of Physics and Electronics, Shandong Normal University, Jinan, 250014 China; 2 College of Chemistry, Chemical Engineering and Materials Science, Collaborative Innovation Center of Functionalized Probes for Chemical Imaging, Key Laboratory of Molecular and Nano Probes, Ministry of Education, Shandong Normal University, Jinan, 250014 People’s Republic of China; 3 State Key Laboratory of Precision Spectroscopy and Department of Physics, Institute of Theoretical and Computational Science, East China Normal University, Shanghai, 200062 China; 4 NYU-ECNU Center for Computational Chemistry at NYU Shanghai, Shanghai, China 200062

**Keywords:** Molecular dynamics, Polarization effect, Protein folding, Solvent model

## Abstract

Molecular dynamic (MD) simulations with both implicit and explicit solvent models have been carried out to study the folding dynamics of HP-36 protein. Starting from the extended conformation, the secondary structure of all three helices in HP-36 was formed in about 50 ns and remained stable in the remaining simulation. However, the formation of the tertiary structure was difficult. Although some intermediates were close to the native structure, the overall conformation was not stable. Further analysis revealed that the large structure fluctuation of loop and hydrophobic core regions was devoted mostly to the instability of the structure during MD simulation. The backbone root-mean-square deviation (RMSD) of the loop and hydrophobic core regions showed strong correlation with the backbone RMSD of the whole protein. The free energy landscape indicated that the distribution of main chain torsions in loop and turn regions was far away from the native state. Starting from an intermediate structure extracted from the initial AMBER simulation, HP-36 was found to generally fold to the native state under the dynamically adjusted polarized protein-specific charge (DPPC) simulation, while the peptide did not fold into the native structure when AMBER force filed was used. The two best folded structures were extracted and taken into further simulations in water employing AMBER03 charge and DPPC for 25 ns. Result showed that introducing polarization effect into interacting potential could stabilize the near-native protein structure.

## Introduction

Prediction of protein’s native structure from its sequence is still one of the greatest challenges in computational biology. Molecular dynamics (MD) simulation, serving as a complementary means to experimental probe, is capable of providing all kinetic and thermodynamic information on protein folding. Due to the limitations in computing power and accuracy of force field, applications of computational investigations of protein folding are restricted to small “fast folders” [1–3]. The villin headpiece subdomain, a 36 residues peptide (HP-36), has served as a benchmark system for the study of protein folding by all-atom molecular dynamics simulations [4–11]. It is one of the smallest proteins that can fold autonomously without the assistance of disulfide bonds, metal ions, or non-natural amino acids. It is thermally stable with the melting temperature over 70 °C in aqueous solution [12]. Its merits of small size, high stability, fast folding kinetics (10–100 μs) [4] make it an ideal prototype for both experimental and computational studies of protein folding [4–6, 13–33]. Several in silico folding and unfolding studies of HP-36 have been carried out [4–7, 21, 27, 34–45] employing either implicit or explicit solvent model. Duan and Kollman [4] performed a 1 μs folding simulation of HP-36 in explicit solvent using AMBER96 force field, from which they proposed a possible folding pathway for this protein. Shen and Freed [5] performed a 200 ns folding simulation for HP-36 using a simple and fast implicit solvent model and found a native-like structure. Hansmann and Wille [42] used a novel heuristic global optimization approach to predict the structure of HP-36 and found a 3D structure very close to the experimental one. Zagrovic et al. [6] used worldwide distributed computing techniques to simulate the folding of the villin headpiece in atomistic detail. Starting from an extended state, they obtained an ensemble of folded structures and the best Cα RMSD was close to 3.8 Å. They found that the folding mechanism of HP-36 was highly consistent with the hydrophobic collapse view of folding. Srinivas and Bagchi [34] performed Brownian dynamics simulation to study the qualitative folding features of HP-36, and they obtained funnel-like energy landscape for protein folding and found a stable native conformation. Fernández et al. [35] revealed three-body correlations in which hydrophobic residues positioned to protect amide-carbonyl hydrogen bonds from attack by water are important in guiding the folding process of HP-36. An ab initio HP-36 folding simulation at 400 K using AMBER force field and GB model for 15 ns by Jang et al. [26] found that the initial hydrophobic collapse and the rapid formation of helices played important roles in the early stage of folding. Also, in this folding simulation, they observed several native-like conformations with the backbone RMSDs from the NMR structure below 4 Å. The simulated free energy profiles indicated that HP-36 adopted two-state thermodynamic behavior. In a molecular dynamics simulation with Poisson-Boltzmann (PB) implicit solvent model and self-guiding force, Wen et al. found that HP-36 folding started with hydrophobic collapse, followed by the formation of helices [39]. Within these efforts, the best sampled conformation of HP36 with a 1.3 Å Cα RMSD for residues 9–32 had been obtained by Jayachandran et al. using worldwide distributed computing techniques [10]. Yang et al. [28] performed a large number of ab initio Monte Carlo folding simulations using a simple transferable all-atom potential [46] for HP-36, and found that the initial collapse was accompanied by the formation of both helix 2 and helix 3, while helix 1 formed at a slower rate. The transition state ensemble in their simulations contained structures with native-like conformations of helix 1 and helix 2. Best optimized AMBER03 force field with a simple correction to the backbone potential, which improved secondary structure balance [47]. With this force field, they found that the villin headpiece reached the folded state in 50 ns and fluctuated about its folded state after the initial folding [33]. Later, they showed that the combination of the optimized AMBER03 force field and TIP4P/2005 superior water model was capable of providing a more accurate description for the solvation of the unfolded state over a wide range of thermodynamics conditions [48]. The new model showed that predicted helical propensities were in better agreement with experiments and yielded a more realistic collapse of unfolded conformations with increasing temperature. Among those studies, the ground breaking folding simulation for 35 residues villin headpiece was first carried out by Shaw group [49], which extended to over 100 μs near the melting temperature in explicit water using the modified CHARMM force field by a specialized supercomputer Anton. Shaw et al. [49, 50] found that the most representative structure of the folded state fell within 2.0 Å RMSD of the native structure. HP-36 had also been used in experimental studies. Hansen et al. [51] used circular dichroism (CD) spectroscopy to monitor the photoinitiated folding of HP-36 in a nondenaturing environment. Wang et al. [16] measured the folding rate (on the order of 10^4^ s^−1^) of HP-36 using dynamics NMR line-shape analysis method. The study found the protein folded on the time scale of 10 μs. A recent experimental study indicated that HP-36 was stabilized by tertiary interactions involving Phe47, Phe51, Phe58, and Val50, and there was significant residual structure in the denatured state of HP-36 which was not due simply to locally stabilized structure [15]. In 2005, experiments with equilibrium Fourier transform infrared (FTIR) and temperature jump (T-jump) IR spectroscopic techniques by Brewer et al. predicted the folding and unfolding times of HP-36 to be 3.34 μs and 6.97 μs respectively at 49.9 °C [17].

HP-36 consists of three short helices (referred to as helix 1, 2, and 3 hereafter) for residues 4 to 8, 15 to 18, and 23 to 30. In between are a loop (residues 9 to 14), a turn (residues 19 to 22), and a closely packed hydrophobic core [4, 12, 52]. In this work, MD simulations were performed to study the folding dynamics of HP-36 starting from an extended conformation in both implicit and explicit solvent models. AMBER03 force field was employed, and the simulations extended to 400 ns. Our results showed that the secondary structure formed on the 50 ns time scale while the tertiary structure was hard to fold in the whole MD simulation. Then the two best folded structures were used as the starting structures for MD simulations in explicit water for 25 ns employing two sets of atomic charges, i.e., AMBER03 charge and the dynamically adjusted polarized protein-specific charge (DPPC). The latter is derived from quantum mechanical calculation for protein using the molecular fractionation with conjugate caps approach [53] combined with the PB solvation model and is updated on-the-fly to account for the dynamic fluctuation of the structure [54, 55]. DPPC includes electronic polarization effect and can provide more accurate electrostatic interactions which has a significant impact on the structure and function of the protein [56]. It had been implemented in several studies, such as pKa of an inner residue in protein prediction [54], secondary structural maintenance [56, 57], NMR J-coupling calculation [58, 59], protein/ligand binding [60], and protein folding [55]. It has been well accepted that one of the limitations in traditional force fields is the lack of explicit electronic polarization, which may lead to failure in protein folding simulation. We found that the two best folded structures were more stable under DPPC than under AMBER charge, indicating the importance of explicit treatment of polarization effect in MD simulation. Our recent work [61], which included the polarization effect in MD simulation, demonstrated the electronic polarization energetically stabilized the helix structure.

## Methods

Starting from linear structure generated by LEaP module in AMBER, two 400 ns MD simulations were carried out with implicit (GB^OBC^ (Onufriev, Bashford and Case model)) [62, 63] and explicit solvent models respectively. AMBER03 force field was employed for both simulations. In the simulation with implicit solvent model, the initial structure was first optimized with steepest descent minimization method for 10,000 steps, and then further relaxed with conjugate gradient method until convergence was reached. The minimized structure was then heated up to 300 K in 100 ps, followed by a MD simulation with a time step of 2 fs. Temperature was regulated using Langevin dynamics [64] with the collision frequency set to 1.0 ps^−1^. The dielectric constants of the protein interior and of the solvent were respectively set to 1.0 and 78.5. Nonbonded interactions were fully counted without any truncations. All the bonds involving hydrogen atoms were constrained by SHAKE algorithm [65] and the salt concentration was set to 0.2 M. The trajectory was saved every 1 ps. In the simulation with explicit solvent model, the protein was placed in a truncated octahedral periodic TIP3P water box with the distance from the surfaces of the box to the closest atoms of the solutes no less than 10 Å. Counterions were added to neutralize the system. Then the system was relaxed in a standard procedure: firstly, only the solvent molecules were free to move, while protein atoms were constrained by an external force; secondly, the whole system was optimized until convergence was reached. After that, the system was heated from 0 to 300 K in 300 ps and then a MD simulation was performed in NPT ensemble to further relax the system without any restraints on solute atoms. Integral time step was set to 2 fs. The Langevin dynamics [64] with a collision frequency of 1.0 ps^−1^ was applied to regulate the temperature. SHAKE algorithm [65] was employed to fix all bonds involving hydrogen atoms. The trajectory was saved every 1 ps and those snapshots were taken in production run for detailed analysis. All the simulations were carried out using AMBER 10 simulation package [66].

Starting from the intermediate structure (with the backbone RMSD of 6.5 Å) obtained in the implicit solvent simulation at 110 ns, another two simulations with both AMBER03 force field and DPPC were further performed using implicit solvent model for 25 ns. DPPC is initially taken from AMBER force field, but atomic charges are periodically updated during the simulation using PPC scheme [54]. The basic procedure for generating PPC can be described as follows: Firstly, to obtain initial electron density of the protein, gas phase quantum mechanical (QM) calculation of protein is performed with the MFCC approach [53]. Secondly, to calculate electrostatic potential of each residue, the restrained electrostatic potential (RESP) procedure is used to fit atomic charges. Thirdly, solution of PB equation is carried out to generate discrete induced charges on the cavity surface. Fourthly, surface charges which are used to mimic the solvation effect are taken as background charges to preform again QM calculation of protein fragment. Finally, the new atomic charges are used again to calculate new solvent induced charges, and the solute and solvent polarize each other until convergence is reached. All QM calculations are performed at B3LYP/6-31G* level. Because PPC is derived on the basis of a single protein structure, for the simulation with a large conformational change, a fixed PPC will bias the simulation. In the current work, we employ a dynamically adapted charge scheme. In principle, updating charges of all atoms at every MD step are indispensable, but it can be prohibitively demanding, due to large computational overhead of QM calculations. Force field parameters other than atomic charges were kept the same as those in AMBER03 simulations.

Then the two best folded structures (with the lowest backbone RMSD) extracted from the trajectories using implicit solvent model and explicit water model were further refined by simulations in explicit water. Both AMBER03 force field and DPPC were utilized. The simulation time was limited to 25 ns and charge updates were carried out every 100 ps in this work to maintain the balance between efficiency and accuracy. The free energy landscapes are determined by calculating the normalized probability employing the weighted histogram analysis method (WHAM) [67–69] from density of state *P(X)* = *exp[−βW(X)]/Z* where *X* is any set of reaction coordinates, *P(X)* is the probability at *X*, and *Z* is the partition function. The relative free energy can be easily expressed as *G(X2)-G(X1) = −RTln[P(X2)/P(X1)]* [70]. QM calculations were carried out by Gaussian 09 [71], and charge updates during the simulations were performed by AMBER 10 with in-house modifications.

## Results and discussion

### Simulation in implicit solvent

#### Folding of individual helix

**Fig. 1:**
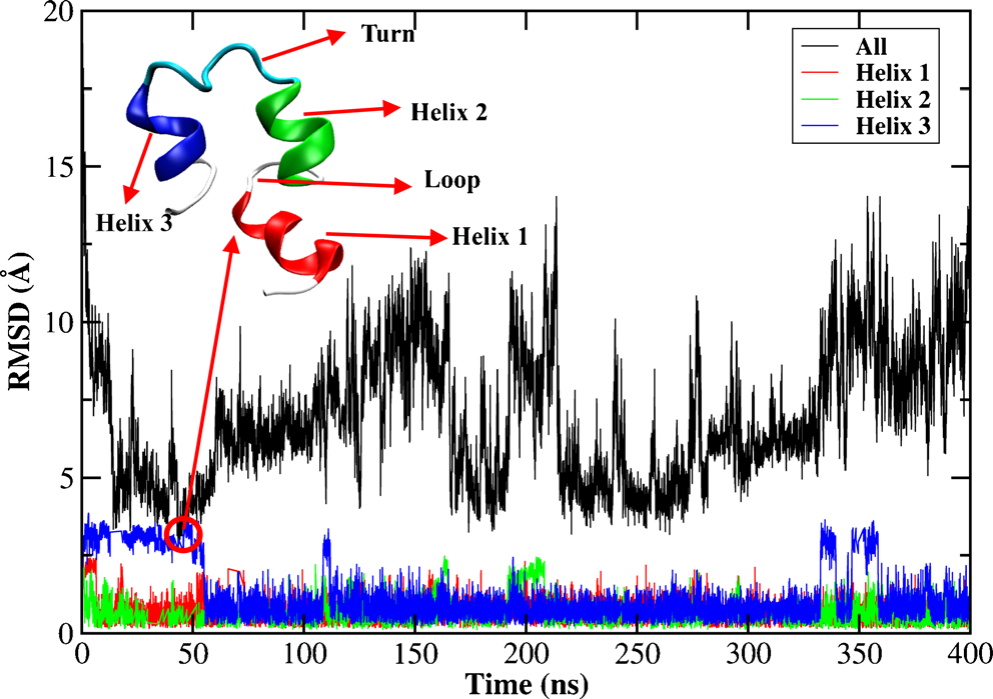
RMSDs of backbone atoms of the HP-36 from the native structure for the whole protein, helix 1, helix 2, and helix 3 as a function of MD simulation time using AMBER03 force field combined with the GB model. The embedded structure is the best folded structure with the lowest backbone RMSD

The backbone RMSDs from the native structure (PDB ID: 1VII) [52] for the whole protein, and for helix 1, helix 2, and helix 3 are shown in Fig. 1. The formation of helix 1 and helix 2 was very fast, which took only 6.5 ns and 1.5 ns respectively. In the remaining time, these two helices remained generally stable with only small fluctuations in RMSD (< 1 Å). However, the formation of helix 3 was much slower (55 ns for the first folding event), but its structure was stable for the rest of the simulation, except that it underwent two unfolding and refolding processes at about 110 ns and 350 ns. RMSD of the whole protein generally fluctuated between 3.5 Å and 10 Å. The best folded structure occurred in 43.5 ns with the lowest RMSD of 2.6 Å, as shown in Fig. 1, which contained three folded helices and a distorted loop between helix 1 and helix 2. Some intermediate folders with the backbone RMSD around 3.5 Å were found in the trajectory from 15 to 50 ns, 175 to 185 ns, and 220 to 270 ns, but these intermediates were not energetically favorable, and the structure quickly drifted away. In contrast, formation of the tertiary structure was very difficult. Folded state, defined by RMSD below 3.5 Å, was rarely seen. Its occurrence was only around 0.45 %. The observation was in good agreement with the experimental observation that the secondary structure could form in the early stage of folding, while the formation of tertiary structure was a much later event [72].

#### The influence of loop and hydrophobic core

**Fig. 2:**
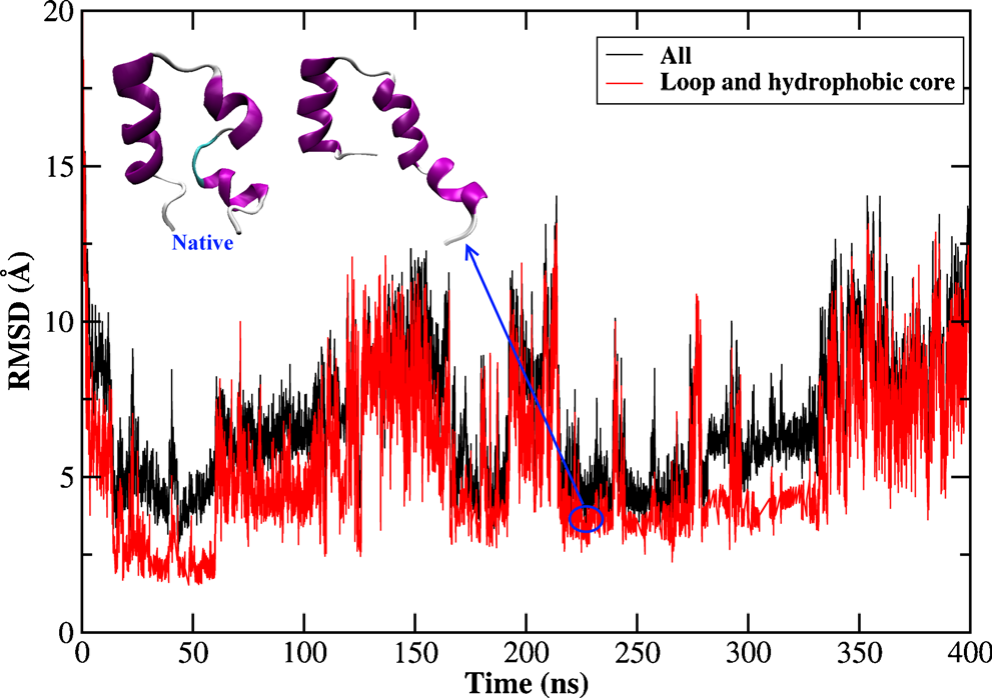
RMSDs of backbone atoms of the HP-36 from the native structure for the whole protein, loop, and hydrophobic core, as a function of MD simulation time using AMBER03 force field and GB model. And the native structure (the left embedded structure) and the representative structure selected from the most populated cluster (the right embedded structure)

A previous study by Duan et al. [21] suggested that the hydrophobic core formed by LYS8, LEU35 and PHE36 played an important role in stabilizing the structure. In our simulation, this hydrophobic core had not been formed. Besides, we noticed that there was a strong correlation between the total RMSD and that of the loop plus hydrophobic core with the correlation coefficient 0.93, as shown in Fig. 2. Although the potential energy was still stable along the entire trajectory demonstrating a well-behaved MD simulation, the loop and hydrophobic core were not formed, which resulted in a violent fluctuation of the total RMSD. The representative conformation of the peptide from the most populated cluster has a backbone RMSD of 4.2 Å from the native structure. Both of them were shown in Fig. 2. It clearly demonstrated that the length of helix 2 was too long, which narrowed the space that could be visited by loop between helix 1 and helix 2, thus hindering the packing of helix 1 over the other two. This phenomenon was also observed by Rajan et al., who found that helix 1 and helix 2 almost merged together and the structure was very stable in the first 3 μs [73].

#### The distribution of backbone torsions in loop and turn regions

**Fig. 3:**
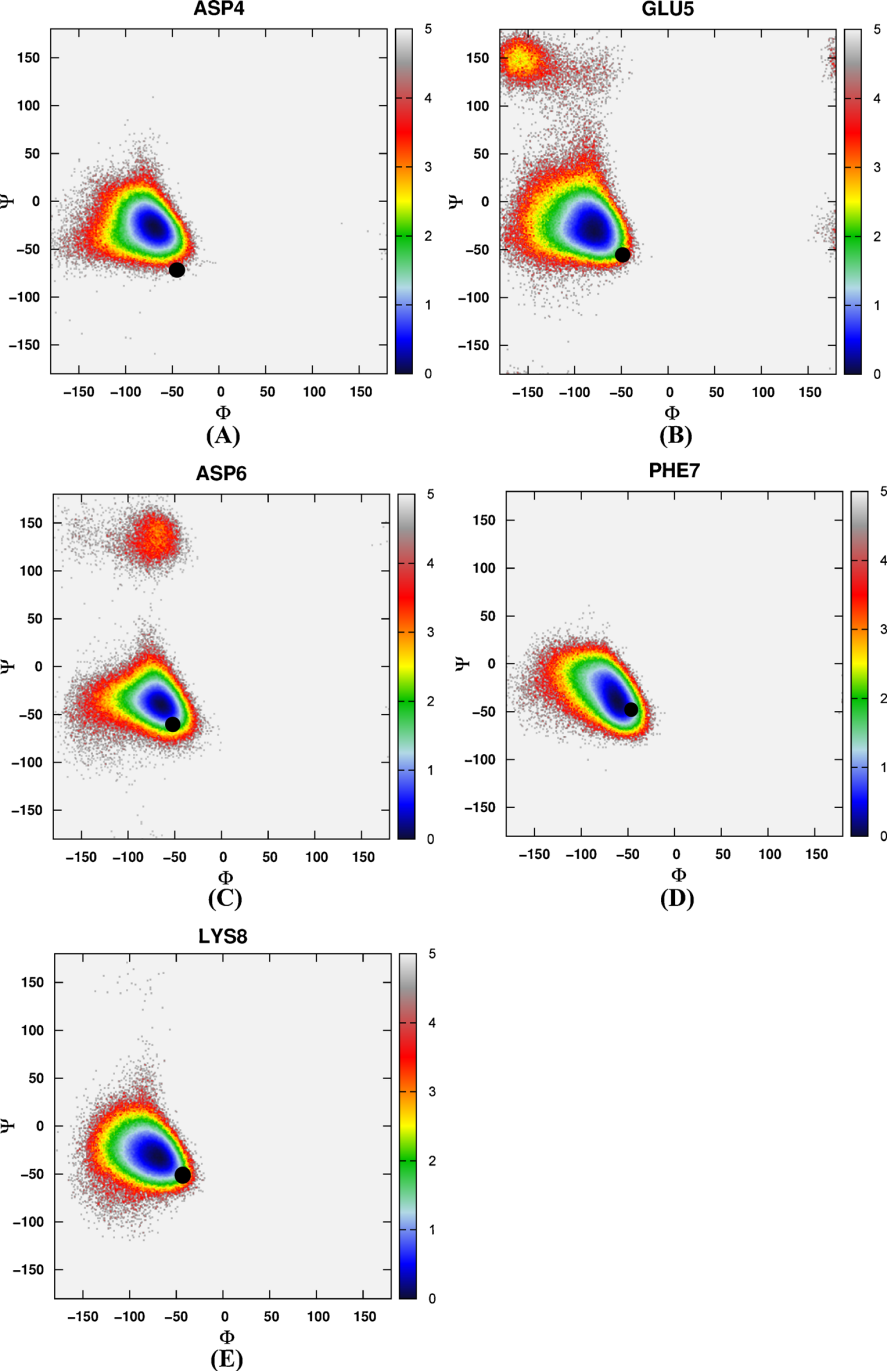

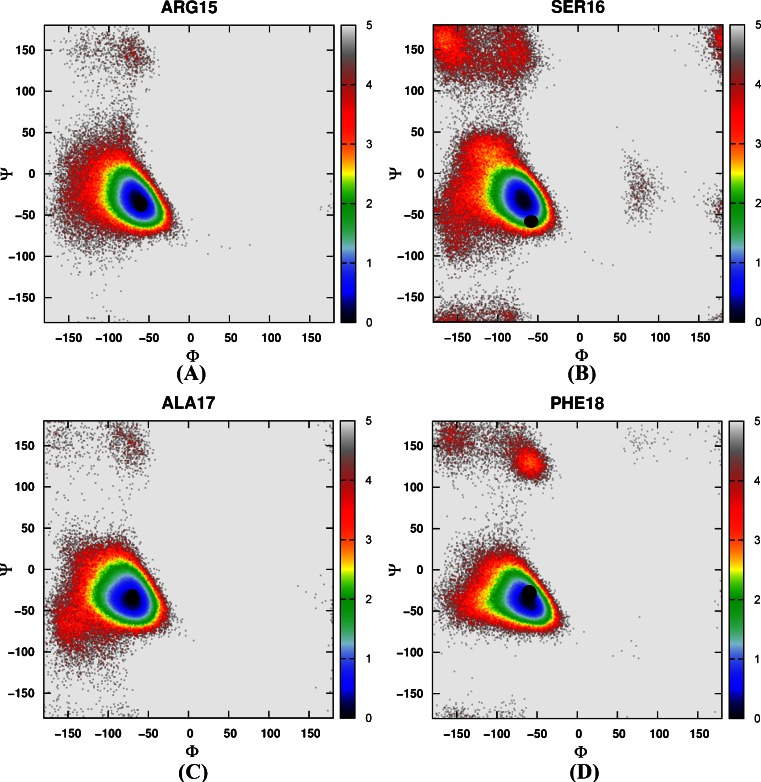

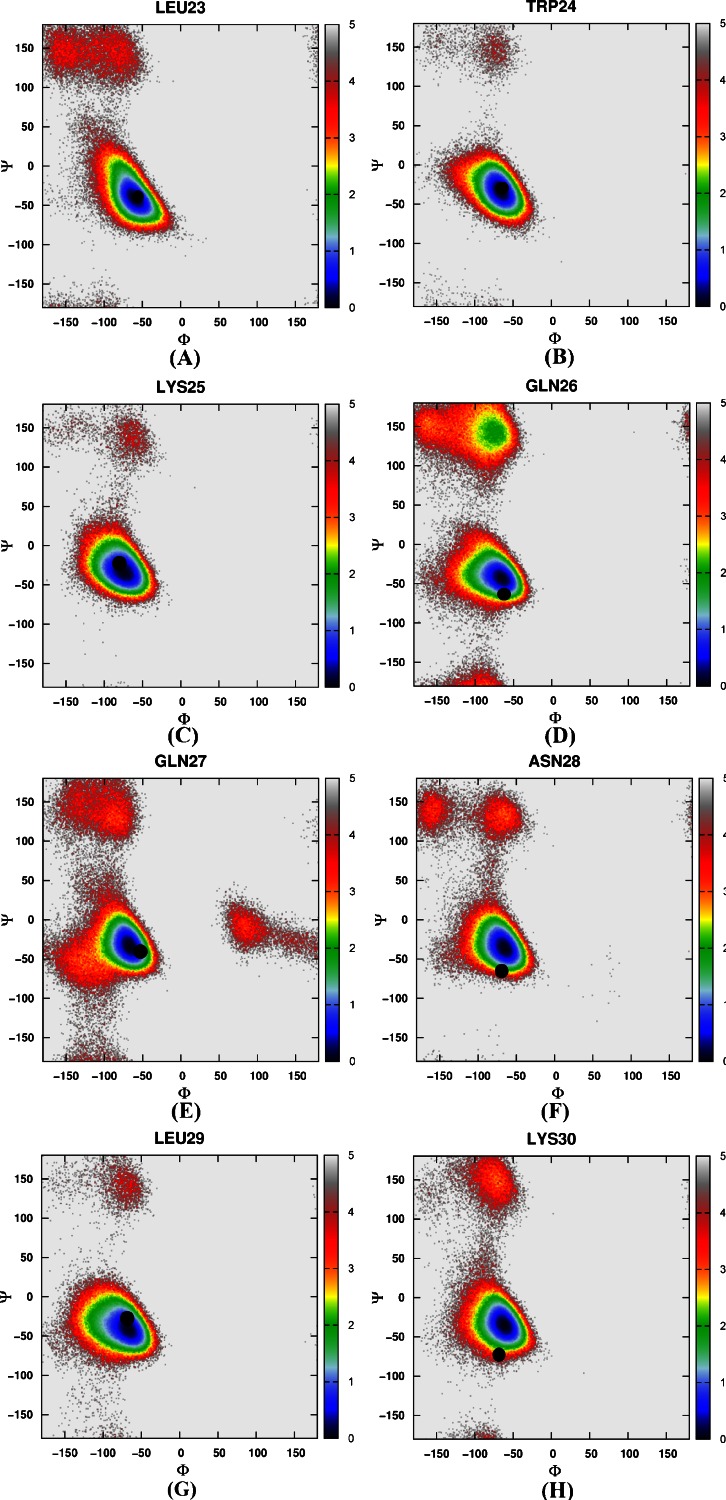
Free energy contour maps as a function of these torsions of helix 1 (**1**) for ASP4 (*A*), GLU5 (*B*), ASP6 (*C*), PHE7 (*D*), LYS8 (*E*), of helix 2 (**2**) for ARG15 (*A*), SER16 (*B*), ALA17 (*C*), PHE18 (*D*), of helix 3 (**3**) for LEU23 (*A*), TRP24 (*B*), LYS25 (*C*), GLN26 (*D*), GLN27 (*E*), ASN28 (*F*), LEU29 (*G*), LYS30 (*H*) using AMBER03 force field and GB model. *Black point* denotes the value of corresponding torsion in native state

**Fig. 4:**
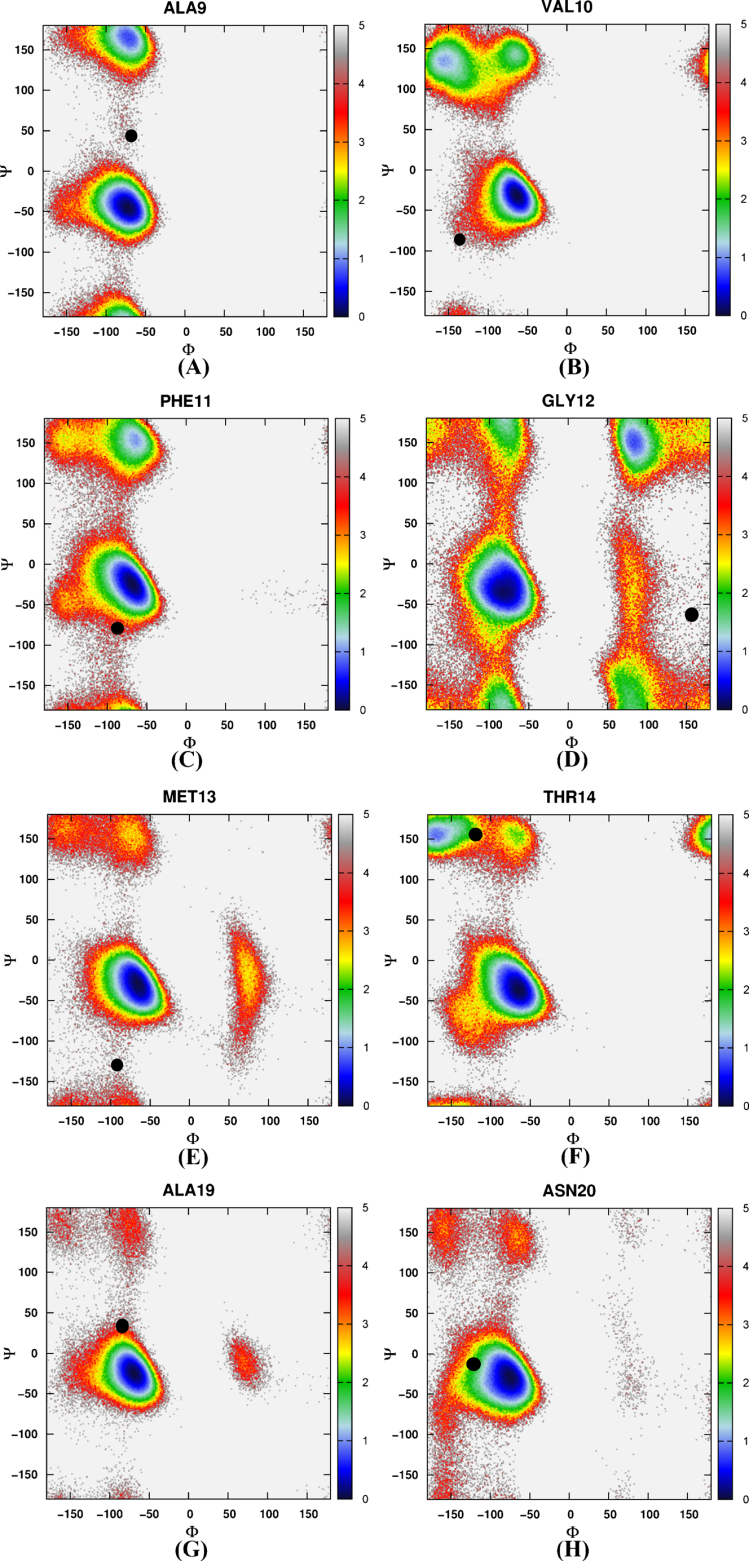
Free energy contour maps as a function of these torsions of loop region for ALA9 (**a**), VAL10 (**b**), PHE11 (**c**), GLY12 (**d**), MET13(**e**) and THR14 (**f**), of turn region for ALA19 (**g**) and ASN20 (**h**) using AMBER03 force field and GB model. *Black point* denotes the value of corresponding torsion in native state

Then the free energy landscapes were constructed by using the main chain φ (C-N-Cα-C) and ψ (N-Cα-C-N) angles, it can be easily found that the distributions of main chain torsions of helix 1, helix 2, helix 3 under MD simulations and NMR structure were very close (see Fig. 3). While the distributions of main chain torsions in turn and loop regions were far away from the native state and the corresponding free energy landscape was shown in Fig. 4. Figure 4a showed the distribution of the main chain torsion of ALA9 located at (φ, ψ) = ((−72˚, −45˚), which was far away from that in the native structure (−79˚, 39˚). This mismatch also applied to residues from VAL10 to THR14 in the loop region (Fig. 4b–f). Those lowest free energy states were far away from those corresponding native states except that the torsion of PHE11 fell within the proximal region of the native state. The distributions of the torsions for ALA19 and ASN20 in turn region were also shown in Fig. 4g–h, of which the local minima were near (−69˚, −22˚) and (−75˚, −27˚). The lowest free energy structure shifted from the native state (−85˚, 28˚) and (−107˚, −15˚) significantly. The distributions of torsions of LEU21 and PRO22 in turn region agreed with the native state.

#### The effect of electrostatic polarization

**Fig. 5:**
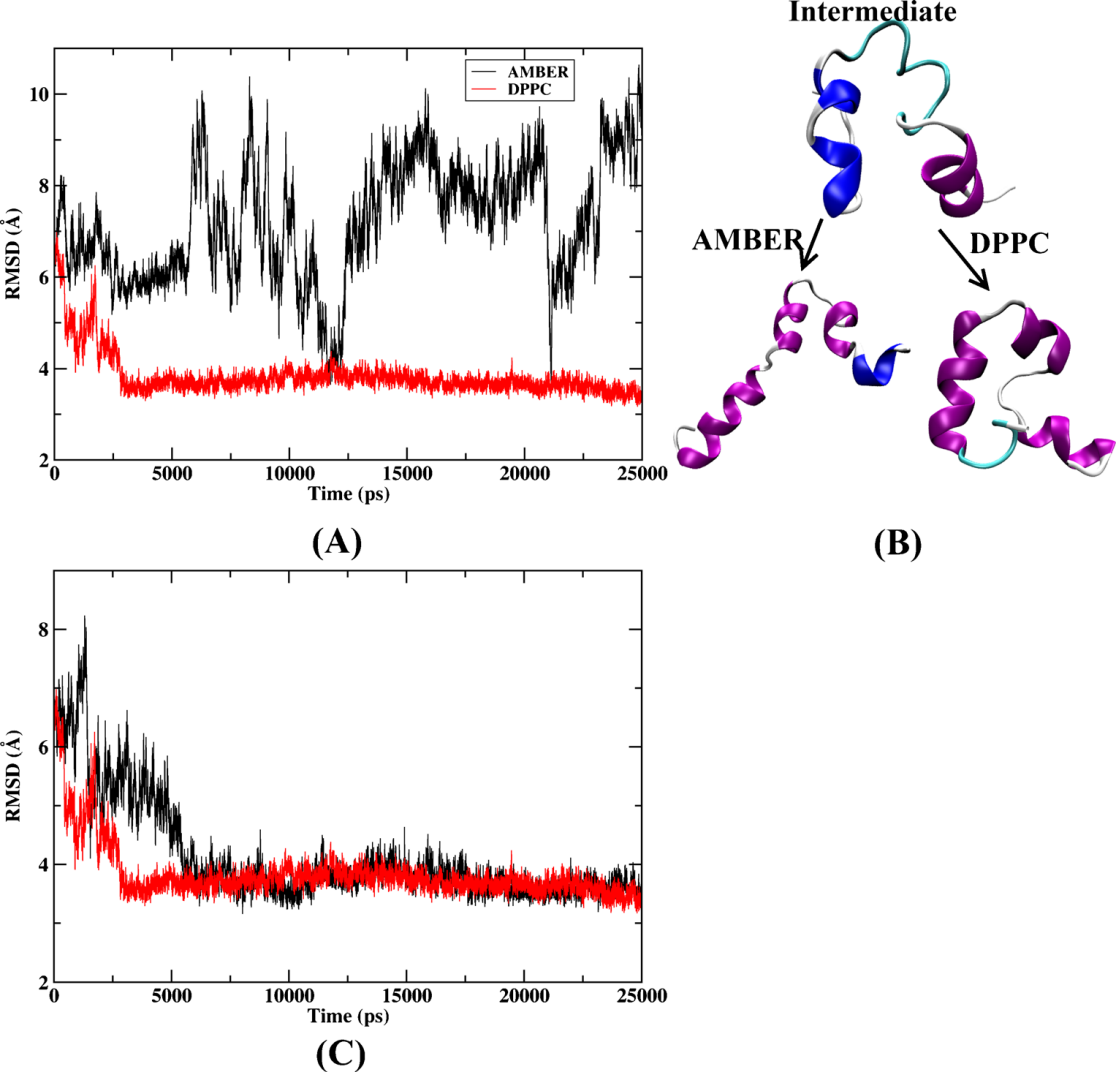
**a** Backbone RMSD of HP-36 from the native structure along MD simulation starting from the intermediate structure (obtained from the implicit solvent simulation) using AMBER03 force field and DPPC combined with the GB model. **b** The intermediate structure and the final structure of MD simulation using AMBER and DPPC, respectively. **c** RMSD of backbone atoms of the peptide as a function of MD simulation time from two MD trajectories. The *red curve* denotes the trajectory discussed in the current paper and the *black curve* denotes another trajectory with the same starting structure but different random seed for momentum

To study the influence of polarization effect on the structure, we started from the intermediate structure (occurred in 110 ns with an RMSD of 6.5 Å) obtained from the above simulation and performed MD simulation with both AMBER03 force field and DPPC for 25 ns using implicit solvent model. The time development of the backbone RMSD was shown in Fig. 5a. Here we use the native structure as the reference structure in the calculation of the backbone RMSD. It can be seen that the peptide did not show evidence of folding toward the native structure under AMBER simulation. The RMSD fluctuation was dominated around 8.0 Å. However, in the DPPC simulation, the RMSD underwent a rapid fall from an initial value of 6.5 Å to around 3.5 Å at about 3 ns and then fluctuated around this value in subsequent MD simulations. This denoted that the protein stayed in the native-like conformation and this was supported by Fig. 5b, which plotted the intermediate structure and two sets of final structures of MD simulation using AMBER and DPPC respectively. In the intermediate structure, helix 2 was not formed and helix 3 was only partially formed. After DPPC simulation, those helices were completely formed and the tertiary structure was generally consistent with the native structure. In comparison, although those helices were formed in the final structure from AMBER simulation, the overall topology was far away from the native state. This result indicated that DPPC simulation can be applied to restore the intermediate structure toward native state of the protein.

It should be mentioned that the result based on single trajectory simulation of DPPC may not be sufficiently used to support our conclusion. So another trajectory was run using DPPC starting from the same intermediate structure but with a different random seed. This is shown in Fig. 5c in which the backbone RMSD was plotted as a function of the simulation time from two DPPC trajectories. As shown in this figure, the latter trajectory reached the folded structure in about 5.5 ns which was slightly longer than in the former DPPC simulation. Although there were some differences in the folding path and steps, the tertiary structure had formed and the folded structure was stable in each individual trajectory.

### Simulation in explicit solvent

#### Folding of individual helix

**Fig. 6:**
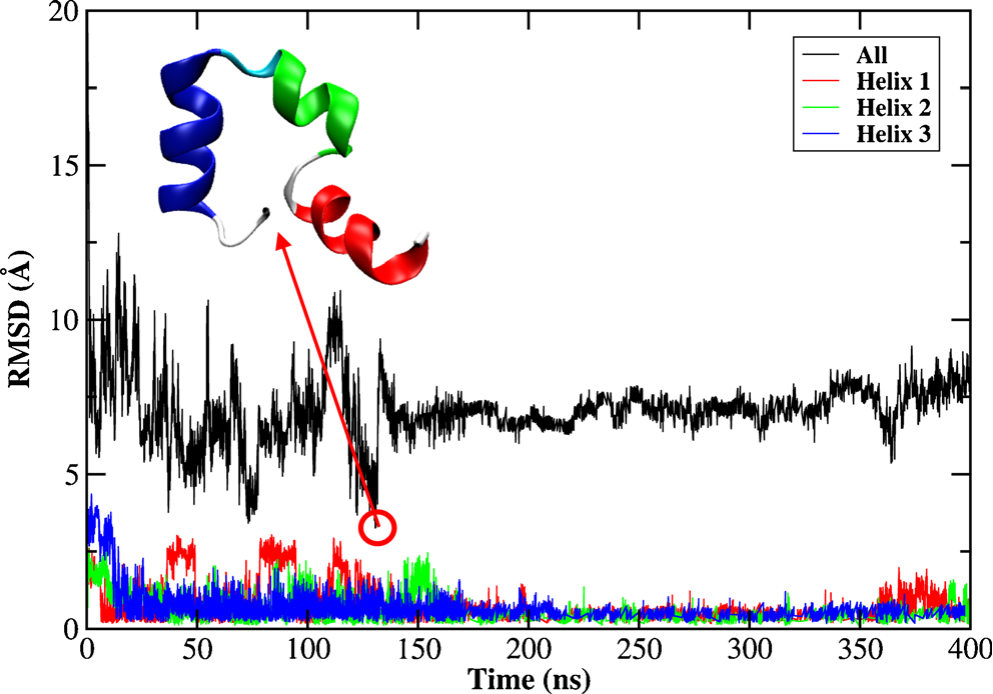
RMSDs of backbone atoms of the HP-36 from the native structure for the whole protein, helix 1, helix 2, and helix 3 as a function of MD simulation time using AMBER03 force field in explicit water model. The embedded structure is the best folded structure with the lowest backbone RMSD

In explicit solvent, the peptide was still in denatured states and the folded structure was scarcely found during the whole MD simulation (400 ns) using AMBER force field. The backbone RMSDs of the whole protein and helices 1 to 3 are shown in Fig. 6. Helix 1 began to form in about 7 ns and helices 2 and 3 were in about 15 ns, but their stabilities were very vulnerable with RMSD fluctuating around 1 ∼ 3 Å before 200 ns and then stabilized in subsequent MD simulation. It could be seen that the backbone RMSD fluctuated at about 7.5 Å and its occurrence was only 0.04 % for which the RMSD was below 3.5 Å, suggesting that the peptide was still completely denatured. It can be seen from the RMSD results that the formation of secondary structures dominated the early stage of folding and the tertiary contacts were still nonspecific, which was in excellent agreement with the experimental observation that nascent secondary structure elements could form in an early stage of folding [72]. It has been shown that simulations with implicit and explicit solvent models can produce different ensembles of structures [74, 75]. It seems that the AMBER03 force field works better with the implicit model for folding the HP-36 than the explicit water model.

#### The influence of loop and hydrophobic core

**Fig. 7:**
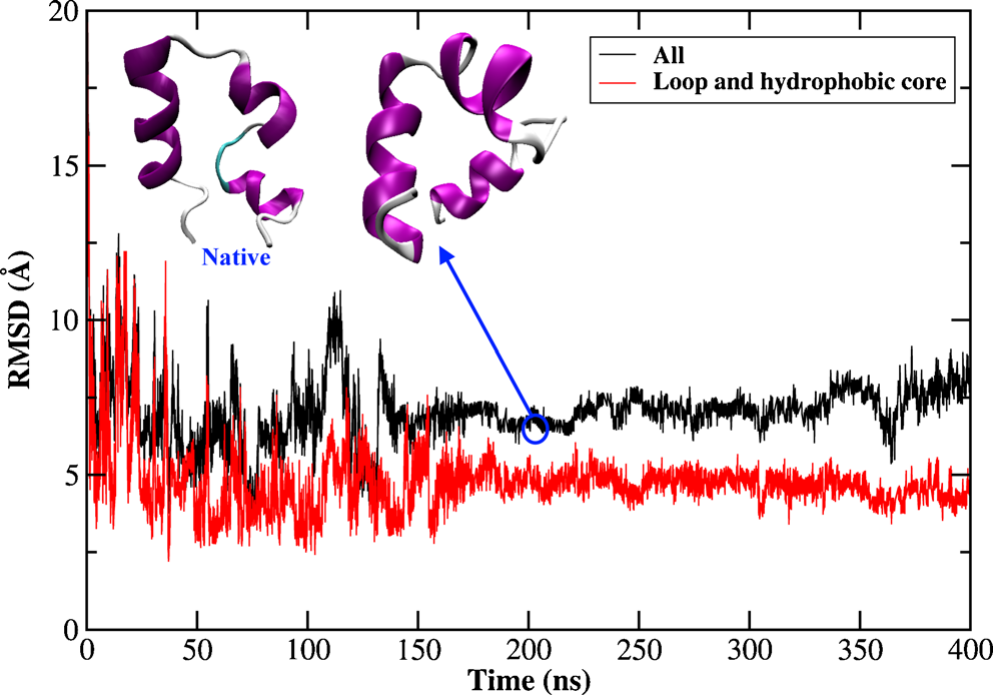
RMSDs of backbone atoms of the HP-36 from the native structure for the whole protein, loop and hydrophobic core, as a function of MD simulation time using AMBER03 force field in explicit water model. And the native structure (the left embedded structure) and the representative structure selected from the most populated cluster (the right embedded structure)

It was noteworthy that the variations of the backbone RMSD of all residues and the backbone RMSD of the loop plus the hydrophobic core, as shown in Fig. 7, were quite similar along the MD trajectory. There was a good correlation with the change of the RMSD of all residues and the loop and hydrophobic core regions. The correlation coefficient and the slope were 0.71 and 0.65 respectively. This indicated that the variations of the loop and hydrophobic core regions had an important impact on the variations of RMSD of all residues, which agreed with the finding in the simulation employing implicit model. The representative structure of the peptide with a backbone RMSD of 7.0 Å selected from the most populated cluster and the native structure were also shown in Fig. 7. Three helices had formed while the loop and hydrophobic core were far away from the native structure in Fig. 7.

#### The distribution of backbone torsions in loop and turn regions

**Fig. 8:**
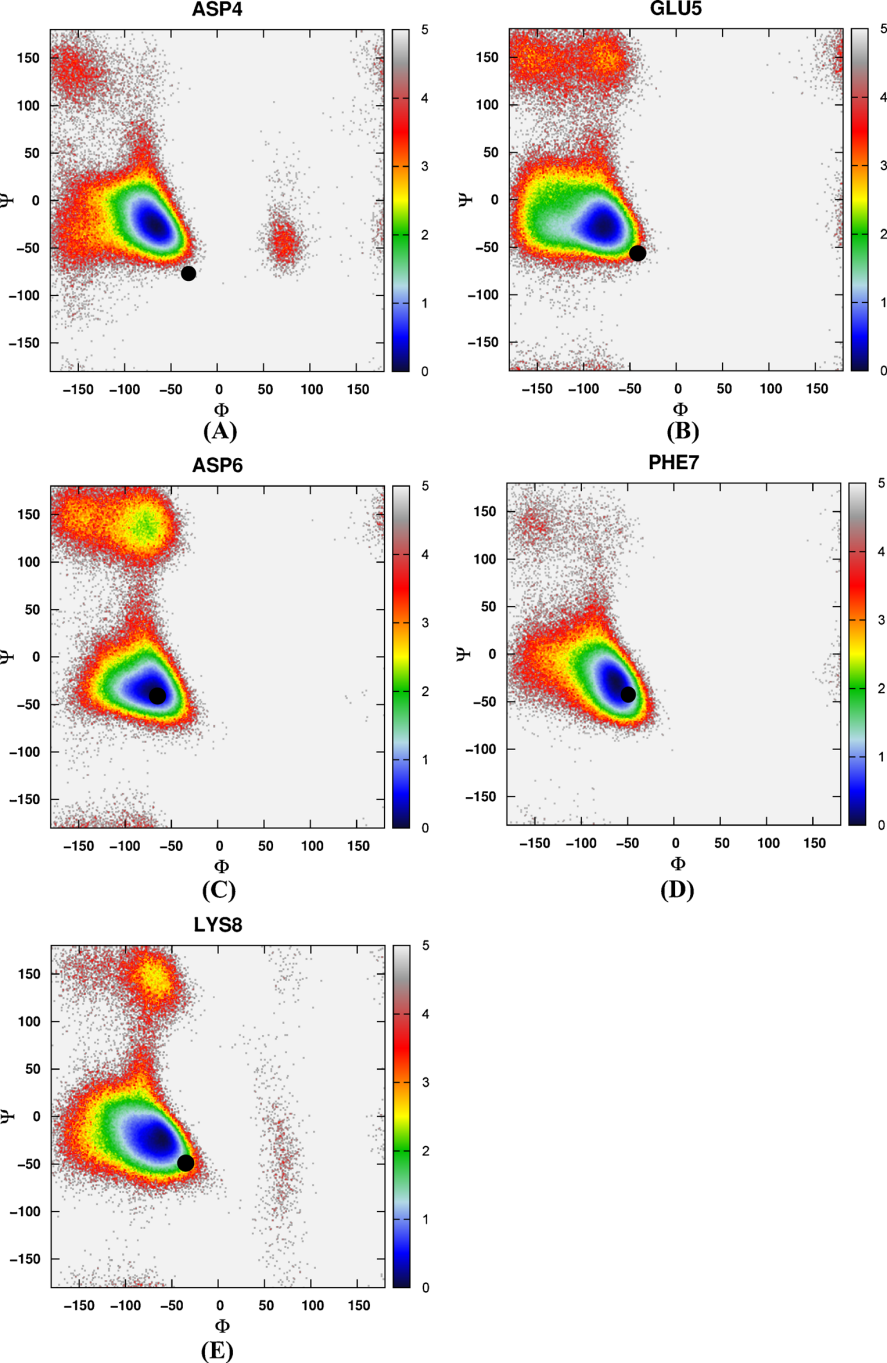

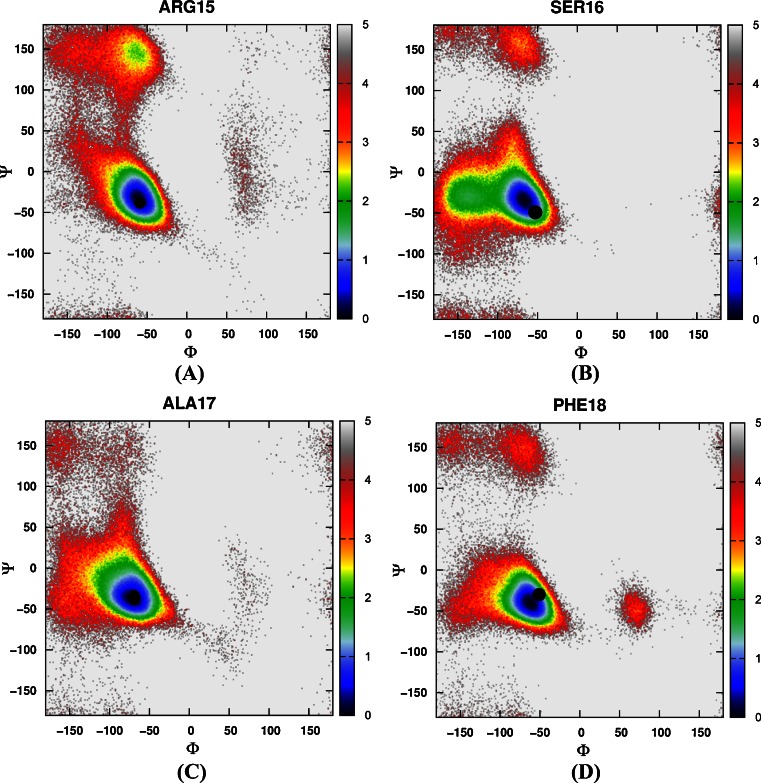

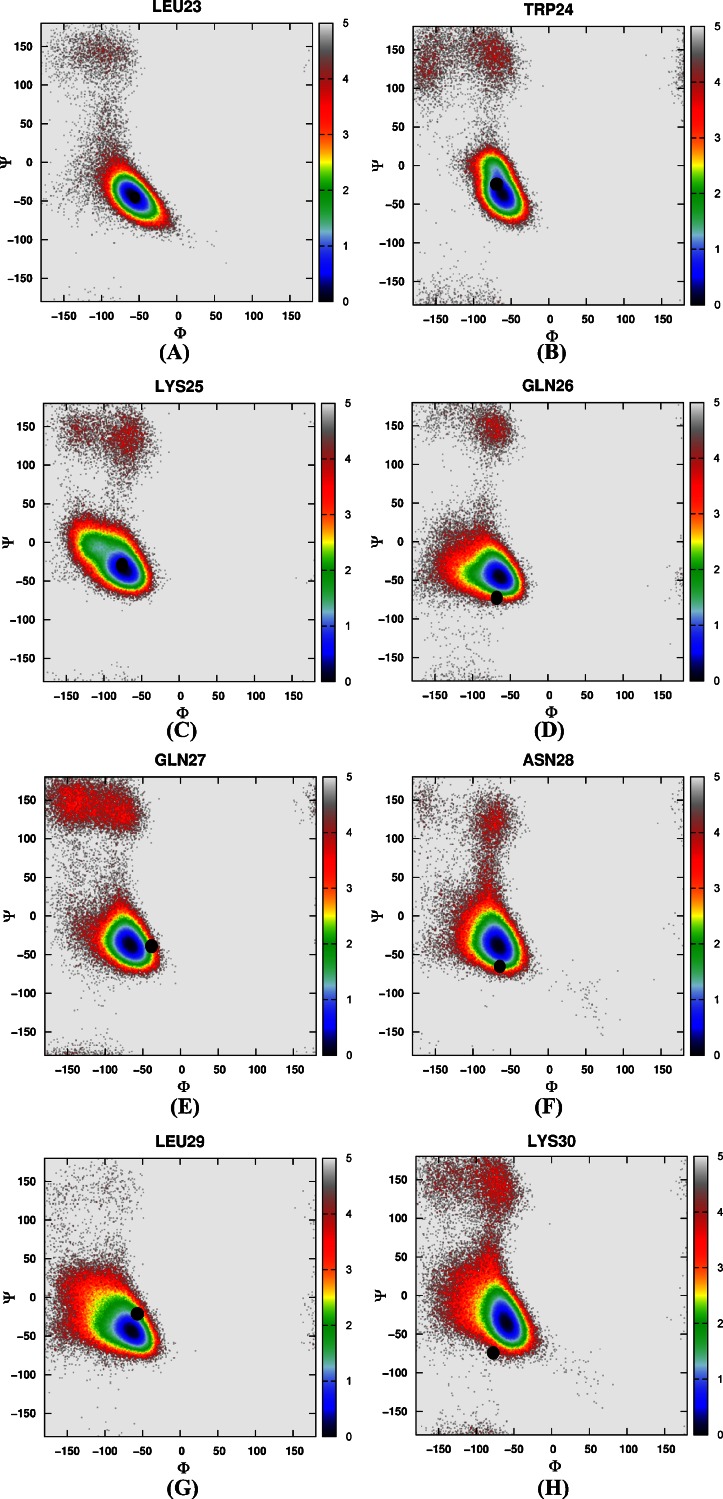
Free energy contour maps as a function of these torsions of helix 1 (**1**) for ASP4 (*A*), GLU5 (*B*), ASP6 (*C*), PHE7 (*D*), LYS8 (*E*), of helix 2 (**2**) for ARG15 (*A*), SER16 (*B*), ALA17 (*C*), PHE18 (*D*), of helix 3 (**3**) for LEU23 (*A*), TRP24 (*B*), LYS25 (*C*), GLN26 (*D*), GLN27 (*E*), ASN28 (*F*), LEU29 (*G*), LYS30 (*H*) using AMBER03 force field and explicit water model. *Black point* denotes the value of corresponding torsion in native state

**Fig. 9:**
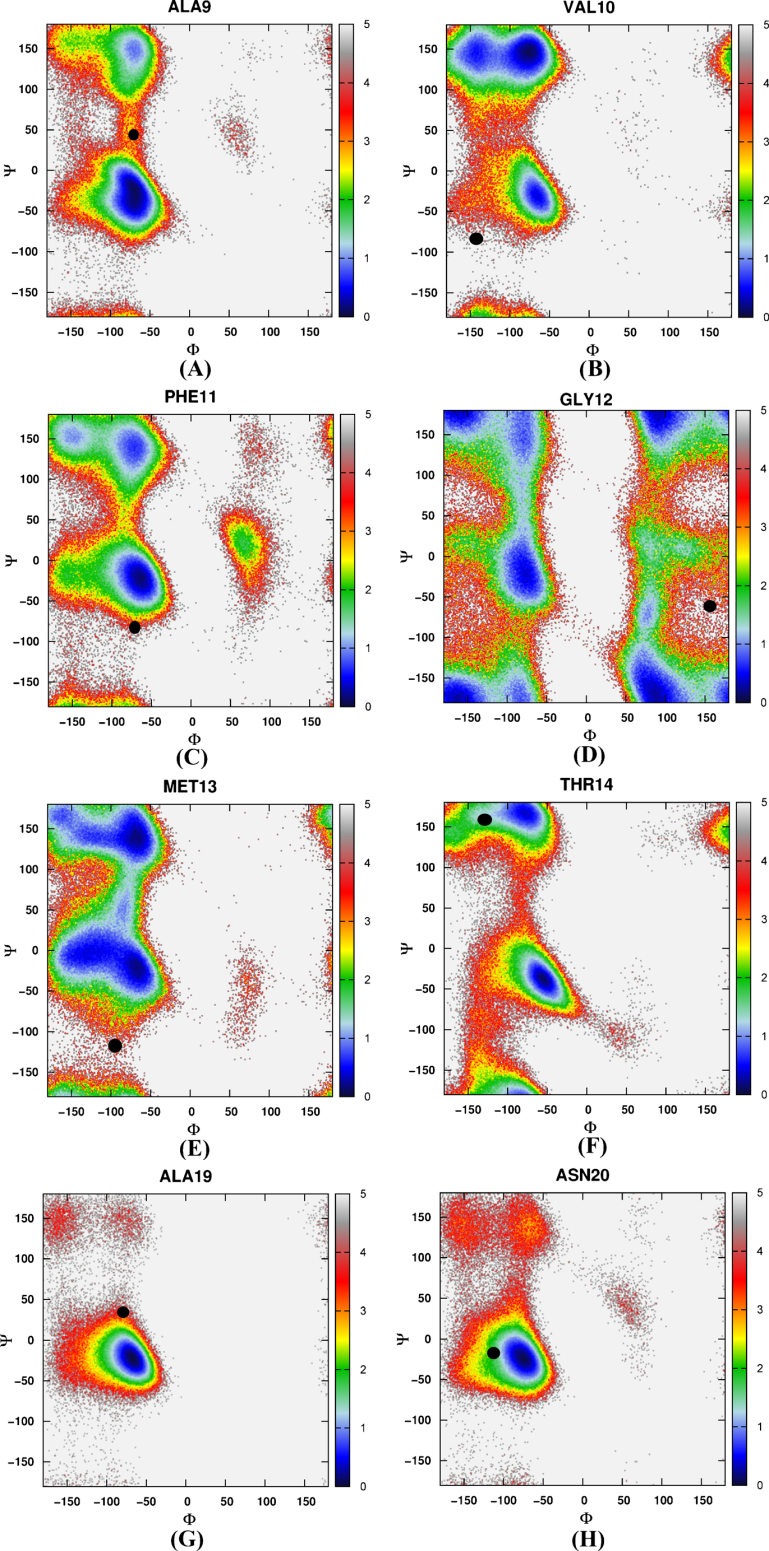
Free energy contour maps as a function of these torsions of loop region for ALA9 (**a**), VAL10 (**b**), PHE11 (**c**), GLY12 (**d**), MET13(**e**) and THR14 (**f**), of turn region for ALA19 (**g**) and ASN20 (**h**) using AMBER03 force field and explicit water model. *Black point* denotes the value of correponding torsion in native state

Again, we constructed a two-dimensional free energy landscape with φ and ψ angles as reaction coordinates. The main chain torsions of helix 1, helix 2, and helix 3 were in excellent agreement with that in the native structure (Fig. 8). However, in the loop and turn regions, the main chain torsions in MD simulation were quite different from that in the native structure (shown in Fig. 9). The calculated free energy minima were far away from the native state, consistent with the implicit solvent model simulation.

#### The effect of electrostatic polarization

**Fig. 10:**
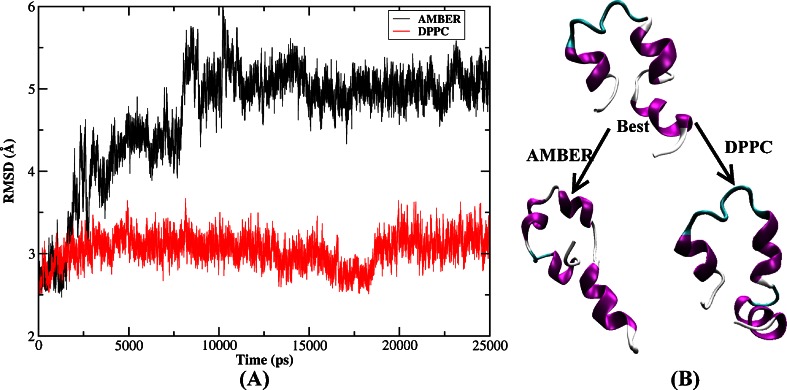
**a** Backbone RMSD of HP-36 from the best folded structure along MD simulation starting from the best folded structure shown in Fig. 1 using AMBER03 force field and DPPC in explicit water (simulation1). **b** The best folded structure and the final structure of MD simulation using AMBER and DPPC, respectively

Another two simulations with explicit solvent model were performed employing both AMBER03 force field and DPPC starting from the best folded conformations obtained using implicit model (denoted simulation1) and explicit water model (denoted simulation2). The best folded structure in implicit model occurred in 43.5 ns with the lowest RMSD of 2.6 Å as shown in Fig. 1. A critical difference of the backbone RMSD for the whole protein (here the best folded structure was the reference structure) can be seen in Fig. 10. Staring from the best folded structure obtained from implicit solvent simulation, the RMSD fluctuated around 3.0 Å using DPPC demonstrating that the structure remained stable in MD simulation. In contrast, the RMSD based on AMBER03 force field was dominated by about 5.0 Å and the average value was 4.7 Å. It was apparent from Fig. 10 that under AMBER force field, the RMSD also visited the vicinity of the folded region with RMSD around 3.0 Å only before 1.5 ns and then it underwent a rapid rise to about 4.0 Å in 1.5 ns to 8 ns in which helices 1 and 3 had very large deformations. Then the RMSD continued to ascend to 5.0 Å at 10 ns in which the tertiary structure was entirely destroyed and helices were only partially formed. Finally the RMSD came to a much more stable stage in which the loop, turn and the hydrophobic core were all destroyed compared with the best folded structure. Figure 10 also plots the comparison between the best folded structure and two sets of final structures resulting from MD simulation using AMBER charge and DPPC, respectively. It was apparent that the structure of DPPC was very close to the best folded structure. However, those tertiary structures were disrupted and the secondary structures also underwent very large deformation in AMBER force field. The current result was another demonstration that the protein structure was stabilized by electrostatic polarization effect, which was in good agreement with previous studies [55, 56, 76]. The computational overhead for DPPC was more expensive than that of AMBER charge, but it was still acceptable. A 500 ps MD simulation took about 12 and 4 h for DPPC and AMBER, respectively, on quad core server.

**Fig. 11:**
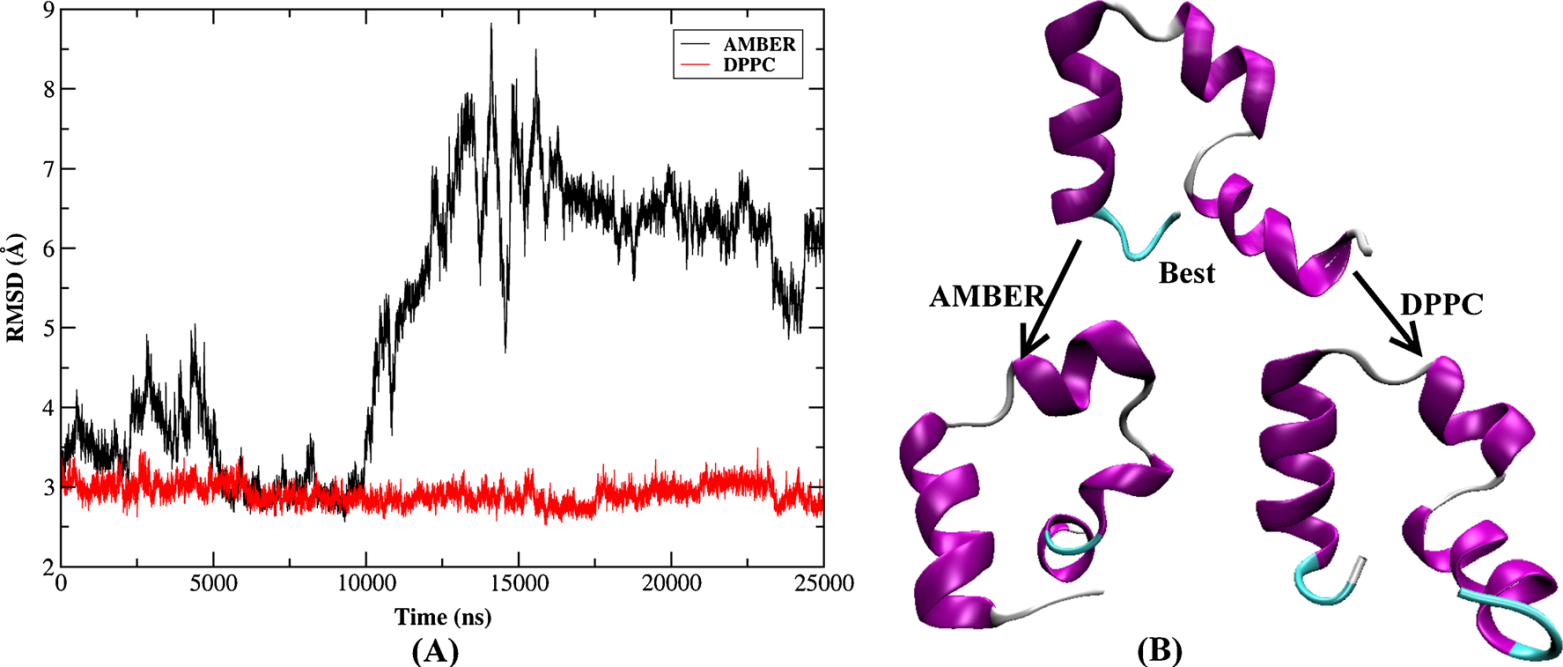
**a** Backbone RMSD of HP-36 from the best folded structure along MD simulation starting from the best folded structure shown in Fig. 6 using AMBER03 force field and DPPC in explicit water (simulation2). **b** The best folded structure and the final structure of MD simulation using AMBER and DPPC, respectively

In explicit solvent model, the best folded structure occurred in 130 ns with an RMSD of 3.1 Å and was also shown in Fig. 6. Then two 25 ns MD simulations were performed in water using this structure as the starting point employing AMBER03 and DPPC. The backbone RMSD for the whole protein along the simulation is shown in Fig. 11. As can be seen, the RMSD was more stable and only fluctuated near 3.0 Å under DPPC simulation. While under AMBER force field, the RMSD fluctuated wildly from 3.0 to 8.0 Å, demonstrating that the peptide was quite unstable. Although some structures were found very close to the native one (with RMSD dropped to 3.0 Å between 5.0 and 9.5 ns), they were not stable and quickly denatured indicating that the instantaneously formed structure was not energetically stable under the standard AMBER force field. The best folded structure and two sets of final structures resulting from MD simulation using AMBER and DPPC were plotted in Fig. 11. It clearly showed that the best folded states were generally stable using DPPC in water model. In AMBER simulation, helix 1 was in a denatured state and helices 2 and 3 retained its secondary structures, whereas the overall topology was far away from the best folded structure.

**Fig. 12:**
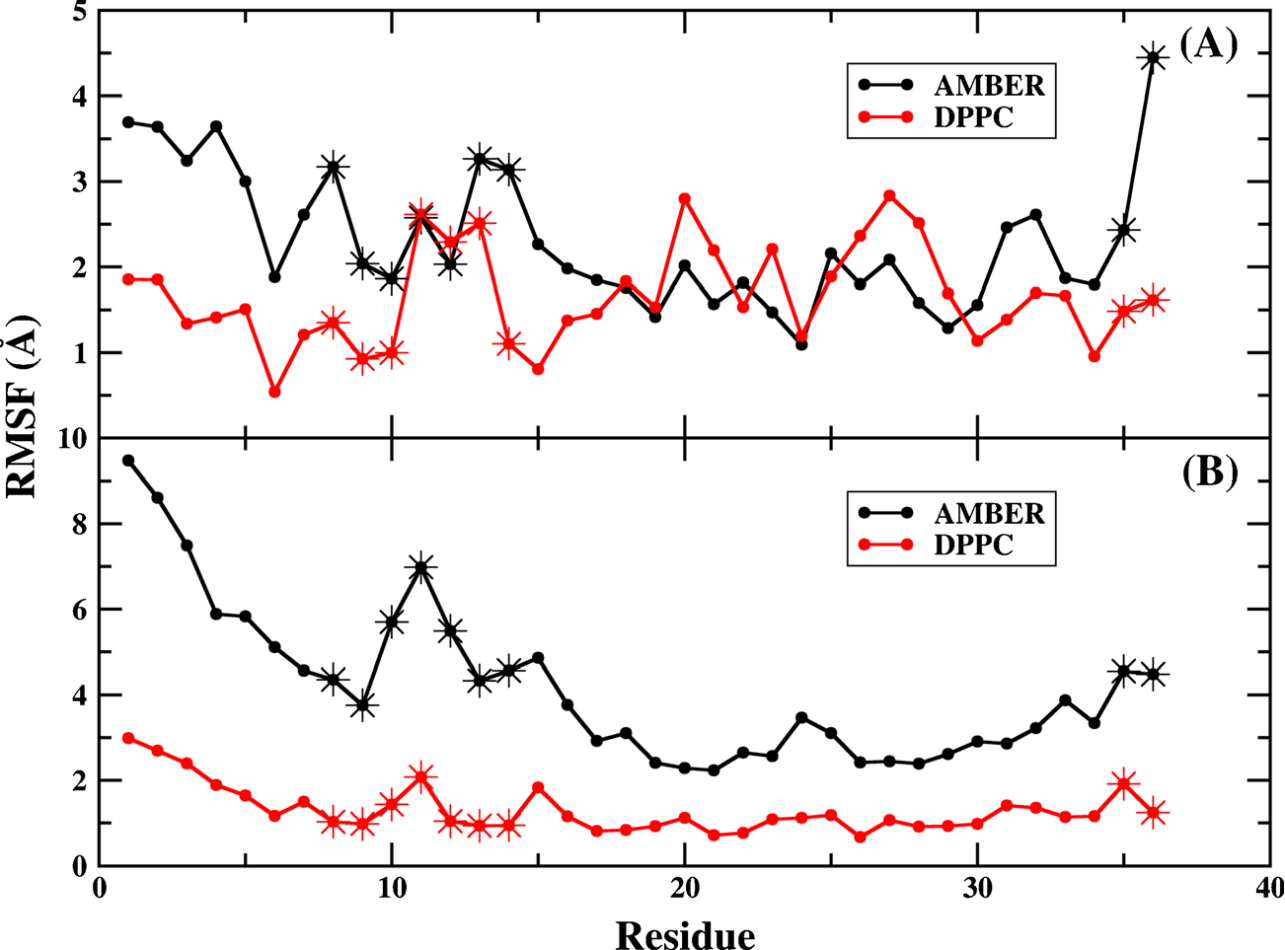
**a** RMSF of the protein starting from the best folded structure obtained using implicit model (simulation1) under AMBER and DPPC simulation **b** RMSF of the protein starting from the best folded structure obtained using explicit model (simulation2) under AMBER and DPPC simulation. *Star* denotes the value of loop and hydrophobic core regions

In order to investigate the motion of loop and hydrophobic core, the root-mean-square fluctuations (RMSFs) of the regions under AMBER charge and DPPC in simulation1 and simulation2 respectively were calculated (shown in Fig. 12). The RMSF reflects the mobility of residue around its average position, so it is also a tool of studying the stability of protein in MD simulation [77]. Obviously, the flexibility of the loop and hydrophobic core regions was higher using AMBER than DPPC in both simulation1 and simulation2. The mean RMSFs were 2.8 and 1.6 Å in simulation1, and they were 4.9 and 1.3 Å in simulation2 for AMBER and DPPC respectively. This suggested that the DPPC performed better in stabilizing the structure of the loop and hydrophobic core. Then, the final structures of two DPPC simulations and the NMR native structure were compared. In simulation1, the distance between LYS8 and LEU35 and that between LYS8 and PHE36 were 9.6 Å, 11.6 Å for the final structure and were 12.0 and 11.6 Å for NMR structure respectively. The final structure had much shorter distance between LYS8 and LEU35 (about 2.4 Å) than that of the native structure and the distance between LYS8 and PHE36 was close to the experimental value. The RMSD of the loop region from the native structure was 1.6 Å. In simulation2, those distances between LYS8 and LEU35, LYS8 and PHE36 were 8.9 and 7.2 Å for the final structure, which was more packed than that for the native state. The RMSD of the loop region from the native structure was 2.9 Å.

**Fig. 13:**
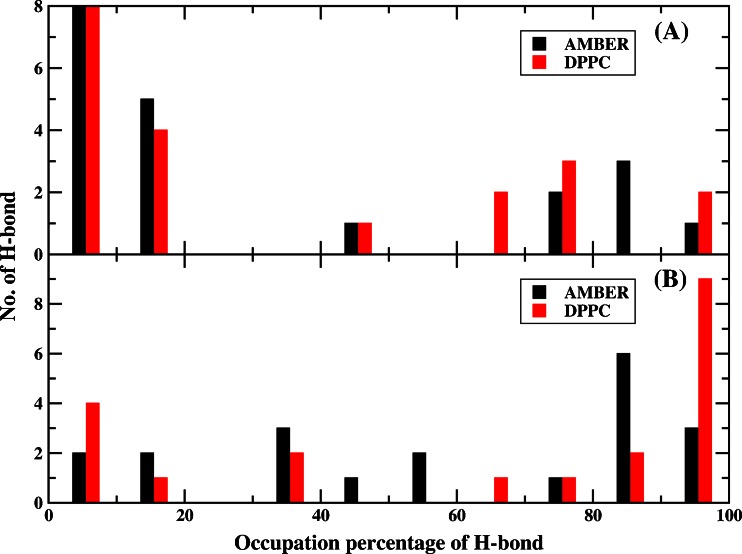
**a** Comparison of the occupation percentage of H-bonds from simulation1 using AMBER and DPPC, respectively. **b** Comparison of the occupation percentage of H-bonds from simulation2 using AMBER and DPPC, respectively

Then we further studied the stability of hydrogen bond and performed statistical analysis of the number of backbone H-bonds to examine the percentage occupancy of them from MD simulation. The percentage occupancy was calculated following the way in ref. [56]. Figure 13 plotted the comparison of H-bonds percentage occupation using a histogram for AMBER and DPPC in simulation1 and simulation2 respectively. Obviously, the distribution of high occupancy H-bonds with DPPC was higher than that with AMBER charge, in good agreement with a previous study [56]. We also noticed that the distributions based on simulation1 using AMBER and DPPC were both dominated by low occupancy H-bonds. Because the initial structure came from the best folded structure (the lowest RMSD) in which helix 3 was not completely formed using implicit model, in the subsequent 25 ns MD simulation in water for AMBER and DPPC, helix 3 was still only partially folded. A recent ab initio folding study for a 17-residues peptide (PDB ID 2I9M) showed that the folding of helix completed in about 80 ns in explicit water model using DPPC [76]. We believe that helix 3 can successfully fold to its native structure after long time DPPC simulation. Besides, our purpose is to examine the importance of polarization effect in stabilizing the near-native protein structure, and it will not affect our conclusion.

**Fig. 14:**
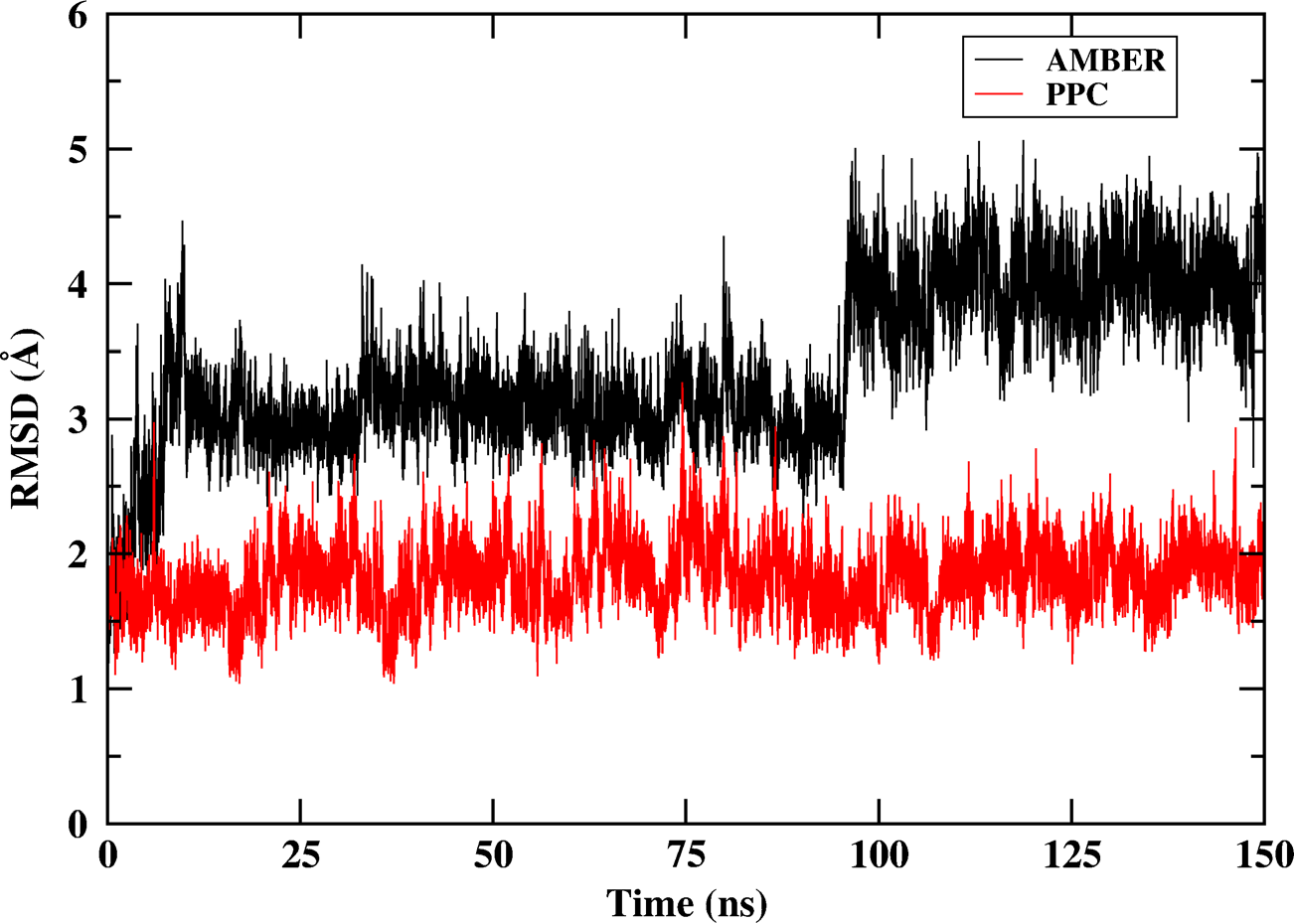
RMSD of backbone atoms of HP-36 from native structure as a function of MD simulation time using standard AMBER and PPC in explicit water model starting from the native state, respectively

To further investigate the polarization effect on the stability of native structure, we carried out MD simulation using AMBER and PPC in explicit solvent model starting from the native structure for 150 ns respectively. In this work, we mainly focused on the dynamic properties of HP-36 near the vicinity of the native state. Thus, we applied PPC scheme in which charge fitting was based on the native structure and was fixed throughout simulation to save the computational expense. As shown in Fig. 14, the native conformation was not stable and the RMSD (from native state) rapidly increased after about 8 ns and fluctuated between 3.0 and 5.0 Å using AMBER. For comparison, the use of PPC was shown to give a more dynamically stable structure with an RMSD of around 1.5 Å. The above result indicated that the native structure was not energetically stable under the standard AMBER force field, which can drive the protein away from its native state. However, the native structure of the protein was stable after long time simulation under PPC. The study demonstrated the important effect of electronic polarization in stabilizing the native structure.

## Conclusions

In this work, folding simulations of HP-36 protein were performed under AMBER03 force field with both implicit and explicit solvent models. Dynamic properties of intermediate structure and the two best folded structures obtained from these simulations were further studied using AMBER03 charge and the dynamically adjusted polarized protein-specific charge (DPPC), respectively. DPPC is derived from a molecular tailoring quantum mechanical calculation of protein in solvent in which the atomic charges of every residue are fitted periodically in the simulation. Our results showed the following features:

1. Under the MD simulation with AMBER03 force field, HP-36 showed ordered secondary structures in about 50 ns starting from an extended structure, while the tertiary structure was hard to reach, regardless of the employed solvent models. It was found that there was a strong correlation between the total RMSD and that of the loop and hydrophobic core regions under the two models.
2. The distributions of main chain torsions of helix 1, helix 2, and helix 3 under two MD simulations were very close to that of the NMR structure. However, in the loop and turn regions, the main chain torsions were quite different from those of the native structure.
3. Starting from an intermediate structure, HP-36 was found to generally fold into the native state under DPPC. In contrast, the peptide did not fold into the native structure with a high RMSD of around 8.0 Å when AMBER force field was used in the MD simulation.
4. When polarization effect was introduced, both of the best folded structures and the native structure were very stable with the low backbone RMSDs in MD simulation. While using AMBER03 force field their stabilities were very low with tertiary structure destroyed and some helices underwent incorrect deformation.
